# Coral reefs in transition: Temporal photoquadrat analyses and validation of underwater hyperspectral imaging for resource-efficient monitoring in Guam

**DOI:** 10.1371/journal.pone.0299523

**Published:** 2024-03-19

**Authors:** Matthew S. Mills, Mischa Ungermann, Guy Rigot, Joost den Haan, Javier X. Leon, Tom Schils

**Affiliations:** 1 Marine Laboratory, University of Guam, Mangilao, Guam; 2 School of Science, Technology, and Engineering, University of the Sunshine Coast, Sippy Downs, Queensland, Australia; 3 PlanBlue GmbH, Bremen, Germany; King Abdulaziz University, SAUDI ARABIA

## Abstract

The island of Guam in the west Pacific has seen a significant decrease in coral cover since 2013. Lafac Bay, a marine protected area in northeast Guam, served as a reference site for benthic communities typical of forereefs on the windward side of the island. The staghorn coral *Acropora abrotanoides* is a dominant and characteristic ecosystem engineer of forereef communities on exposed shorelines. Photoquadrat surveys were conducted in 2015, 2017, and 2019, and a diver-operated hyperspectral imager (i.e., DiveRay) was used to survey the same transects in 2019. Machine learning algorithms were used to develop an automated pipeline to assess the benthic cover of 10 biotic and abiotic categories in 2019 based on hyperspectral imagery. The cover of scleractinian corals did not differ between 2015 and 2017 despite being subjected to a series of environmental disturbances in these years. Surveys in 2019 documented the almost complete decline of the habitat-defining staghorn coral *Acropora abrotanoides* (a practically complete disappearance from about 10% cover), a significant decrease (~75%) in the cover of other scleractinian corals, and a significant increase (~55%) in the combined cover of bare substrate, turf algae, and cyanobacteria. The drastic change in community composition suggests that the reef at Lafac Bay is transitioning to a turf algae-dominated community. However, the capacity of this reef to recover from previous disturbances suggests that this transition could be reversed, making Lafac Bay an excellent candidate for long-term monitoring. Community analyses showed no significant difference between automatically classified benthic cover estimates derived from the hyperspectral scans in 2019 and those derived from photoquadrats. These findings suggest that underwater hyperspectral imagers can be efficient and effective tools for fast, frequent, and accurate monitoring of dynamic reef communities.

## Introduction

Shallow-water tropical reefs are the most biodiverse marine ecosystems on the planet [1]. They are often of significant ecological, economic, and cultural importance to people who depend on them for coastal protection, food, sustenance, tourism, and more [2, 3]. Benthic community composition plays a major role in ecosystem functioning [4] and influences several factors, including species richness [5], recovery from disturbances, and resilience to the effects of climate change [6]. Globally, the health of tropical reefs continues to decline due to chronic and acute stressors such as coastal development, habitat destruction, deforestation, overfishing, destructive fishing practices, pollution, climate change, ocean acidification, sea level rise, and eutrophication [7–15]. These stressors disrupt and negatively impact the community dynamics of tropical reefs [16] and have challenged their ecological functioning [17].

Scleractinian corals are pivotal to the structure and ecology of reef ecosystems, significantly influencing habitat structure [18], carbon fixation, nutrient cycling [19, 20], and reef accretion [21, 22]. Coral bleaching, climate change, and other disturbances have resulted in extensive coral loss, new species configurations, and benthic homogenization [23, 24]. The significant decline of coral health and cover on reefs have been linked to declines in other reef taxa [25], as well as disruptions in food webs, declines in fisheries productivity [26], and destabilization of nutrient cycling [27]. Further, the decline in live corals inevitably leads to ‘coral reef flattening’ [28], or the reduction of habitat structure through the decomposition of coral and erosion of the reef [29]. Repeated disturbances and mass coral bleaching events can exacerbate existing pressures, suppressing coral cover and potentially leading to the collapse of reef ecosystems [30].

The cumulative impact of disturbance events have caused rapid and pronounced changes on coral reefs, many of which do not completely recover and instead undergo a transition (phase shift) to an alternative stable state [31–33]. The best-known phase shifts on coral reefs are transitions from scleractinian-dominated communities to a dominance of fleshy macroalgae [33], though other transitions to communities dominated by coralline or peyssonnelioid algae [34, 35], sponges [36, 37], corallimorphs [38], or other zoantharians [39, 40] have also been reported. Shifts in community composition and overall reef degradation could drive local or regional extinctions of reef-associated species, emphasizing the need to examine reef dynamics at a local scale [41]. The interdependency between ecosystems suggests that a drastic change in one system, such as shallow-water tropical reefs, could have severe consequences for adjacent systems, such as lagoons, seagrass meadows, and reef flats that depend on them for shelter and protection [42].

Rapid and drastic changes of tropical reefs and their negative impact on other ecosystems have compelled researchers to explore new, faster, and more efficient methods to monitor reef communities. Monitoring programs are designed around tradeoffs of scale, time, and accuracy, ranging from accurate but time-consuming field studies to coarser and often less accurate landscape-scale monitoring. Benthic biodiversity and community composition are often characterized using photoquadrat methods [43, 44]. However, the analysis of photoquadrats is an expensive and laborious process and typically covers a tiny fraction of the reef communities of interest. In addition, the significant amount of post-processing and identification time needed to analyze the images suggests that photoquadrat surveys are not ideal when surveying large areas [45, 46]. To meet the needs for larger-scale, more comprehensive, and taxonomically refined monitoring efforts, a number of alternative approaches to speed up benthic surveys have been developed. Remote sensing has proven an effective means of mapping [47–49] and monitoring [50–52] shallow nearshore reefs at high resolution (down to cm scale) [53–55].

The advances in imaging, remote sensing, and other data collection technologies produced massive spatial and temporal datasets and archives, the extent of which were previously unattainable [56]. These large datasets drove researchers to explore novel approaches to efficiently process, analyze, and model them. The application of machine learning is an effective method of analyzing large datasets, and it has been considered a prime candidate for use in ecological studies [57, 58]. In an ecological context, machine learning can be defined as the implementation of computational techniques and the development of dynamic algorithms to identify structure and generate models of complex, nonlinear, and highly dimensional datasets [56, 59, 60]. There are, however, a number of limitations and considerations associated with the use of machine learning. Some of the most frequently observed limitations observed in ecological studies include (1) background complexity masking regions of interest [58]; (2) difficulty validating machine learning models [60]; (3) uncertainty of the applicability of models across geographic regions [61]; and (4) the lack of suitable data needed to effectively employ machine learning techniques [62]. Moreover, machine learning methods typically require a substantial amount of data to train the models [58, 63]. As most biological communities contain rare taxa, the scant representation of these taxa in surveys could affect their identification success using machine learning. Despite this, machine learning methods have produced positive results in a diverse range of ecological applications including biomonitoring, biogeography, conservation and management planning, predictive modelling, hazard assessment, species identification, and habitat mapping in both terrestrial [62, 63] and aquatic [61, 64] environments. While the availability of sufficiently large datasets remains relatively limited for coral reefs, the use of machine learning could also have the potential to rapidly expand the scale of coral reef monitoring beyond a small number of frequently studied areas.

In cases where traditional RGB and multispectral imaging suffered when differentiating between complex and heterogenous reef communities, hyperspectral imaging showed potential to overcome these issues [65–67]. Hyperspectral imaging initially demonstrated the capacity to discriminate benthic components of reefs based on modelled or simulated data [65, 66]. Studies have since utilized hyperspectral data collected from satellite/aerial imagery [68–71], *ex-situ* in a lab setting [72], and underwater [73] to investigate reef communities and the interactions between organisms. Applications of hyperspectral imaging on reefs have ranged from mapping coral cover [68] and examining coral-algal interactions [72], to acting as a bio-optical taxonomic tool based on species-specific absorption signatures [73]. The classification and recognition of benthic communities is the primary application for which satellite, aerial, or underwater hyperspectral imaging is used. Satellite and aerial imagery have shown promise when classifying reefs at community [70, 71] and organismal [66, 67] scales. Nevertheless, as is the case with all imaging and remote sensing methods, there have been limitations associated with the use of hyperspectral imaging on reefs such as the need to account for depth, turbidity, and the optical properties of water [69]. Moreover, large pixel sizes (lower spatial resolution) and spectral mixing have, at times, made it difficult to distinguish organisms in heterogenous habitats [66–70]. Recently, however, the development of close-range underwater hyperspectral imaging has allowed researchers to predict and map reef communities at cm scale [74].

While hyperspectral imaging was initially expensive and computationally challenging to process [65], machine learning has presented a more efficient and cost-effective approach to processing hyperspectral data [75]. Recognizing this potential, a diver-operated underwater hyperspectral imager (HyperDiver) was developed by the Max Planck Institute (Bremen, Germany) to survey and map benthic communities [74]. The HyperDiver is an integrated system that captures color and hyperspectral imagery and various other environmental variables to map benthic communities automatically and efficiently through the implementation of machine learning algorithms. However, the benthic habitat map produced in the initial study from Chennu et al. [74] was only generated for the transect used to train the machine learning algorithms and was not extrapolated to other transects. In addition, the benthic categories consisted predominantly of scleractinian coral taxa and a few broadscale functional groups. Other significant contributors to the benthic communities, including crustose calcifying red algae, macroalgae (red, green, and brown), sponges, cyanobacteria, and hydrozoans, were largely omitted. From 2017 onwards, PlanBlue GmbH (Bremen, Germany) developed the DiveRay with the aim of increasing the scope and utility of underwater hyperspectral imaging. In 2019, the DiveRay was used to evaluate the utility of a diver-operated hyperspectral imager for benthic surveys in Guam.

Guam is the largest and southernmost island of the Mariana Archipelago (13°28’N, 144°46’E). Located just outside of the Indo-Pacific center of reef biodiversity [76], Guam’s mangroves, seagrass beds, lagoons, fringing reefs, and patch reefs are among the most diverse and species-rich nearshore marine ecosystems of all US jurisdictions. Home to more than 350 scleractinian coral species, over 1,000 species of fish, well over 400 seaweed species [77–80], and more than 5,000 documented marine species, Guam’s reefs are among the best-studied in Micronesia [80]. Like other islands in the tropical Pacific, Guam’s reefs and marine resources are deeply rooted in the local traditions and culture and have maintained significant economic value to the present day. The infrastructure protection, fisheries, and recreational value of Guam’s reefs is estimated at roughly $127 million per year [81]. The health of Guam’s reefs, however, has steadily declined since the 1960s [82] with an accelerated loss in scleractinian corals since 2013 [83, 84].

Thermal stress-mediated mass bleaching events have become more prominent since the 1980’s and were first documented in Guam in 1994 [85]. During the island-wide bleaching event of 1994, 68% of coral taxa showed signs of bleaching but mortality was low [85]. No large-scale coral bleaching events were reported for two decades after the 1994 event [82]. Since 2013, however, a succession of environmental disturbances have severely impacted Guam’s reefs. Anomalously high sea surface temperatures (SSTs) caused devastating island-wide bleaching events in 2013, 2014, 2016, and 2017, while a major El Niño-Southern Oscillation (ENSO) event in 2014 and 2015 resulted in large-scale coral die-off due to extreme low tides [84]. In 2013 and 2014, SSTs exceeded the maximum monthly mean over eight consecutive months, resulting in accumulated heat stress reaching peaks of 12 and 9 degree heating weeks (DHW), respectively [83, 84]. The 2016 event was less severe, with accumulated heat stress peaking at 5.5 DHW [84]. However, the event in 2017 was the highest recorded in Guam’s history, where SSTs exceeded the maximum monthly mean over six consecutive months, accumulated heat stress reached a peak of 13 DHW, and corals were observed bleaching at depths of over 30 m [84]. These events caused an estimated 34–37% decline in total coral cover [84, 86]. Habitat-structuring staghorn *Acropora* corals were found to be particularly affected, having suffered upwards of 53 ± 10% mortality [83, 84]. This study aims to precisely document the change in benthic community composition from 2015–2019 at Lafac Bay, initially a pristine site with a high standing stock of staghorn *Acropora* species. In addition, the application of the diver-operated hyperspectral imager (DiveRay) for automated benthic community characterization and monitoring is evaluated.

## Materials & methods

### Study site

**Fig 1:**
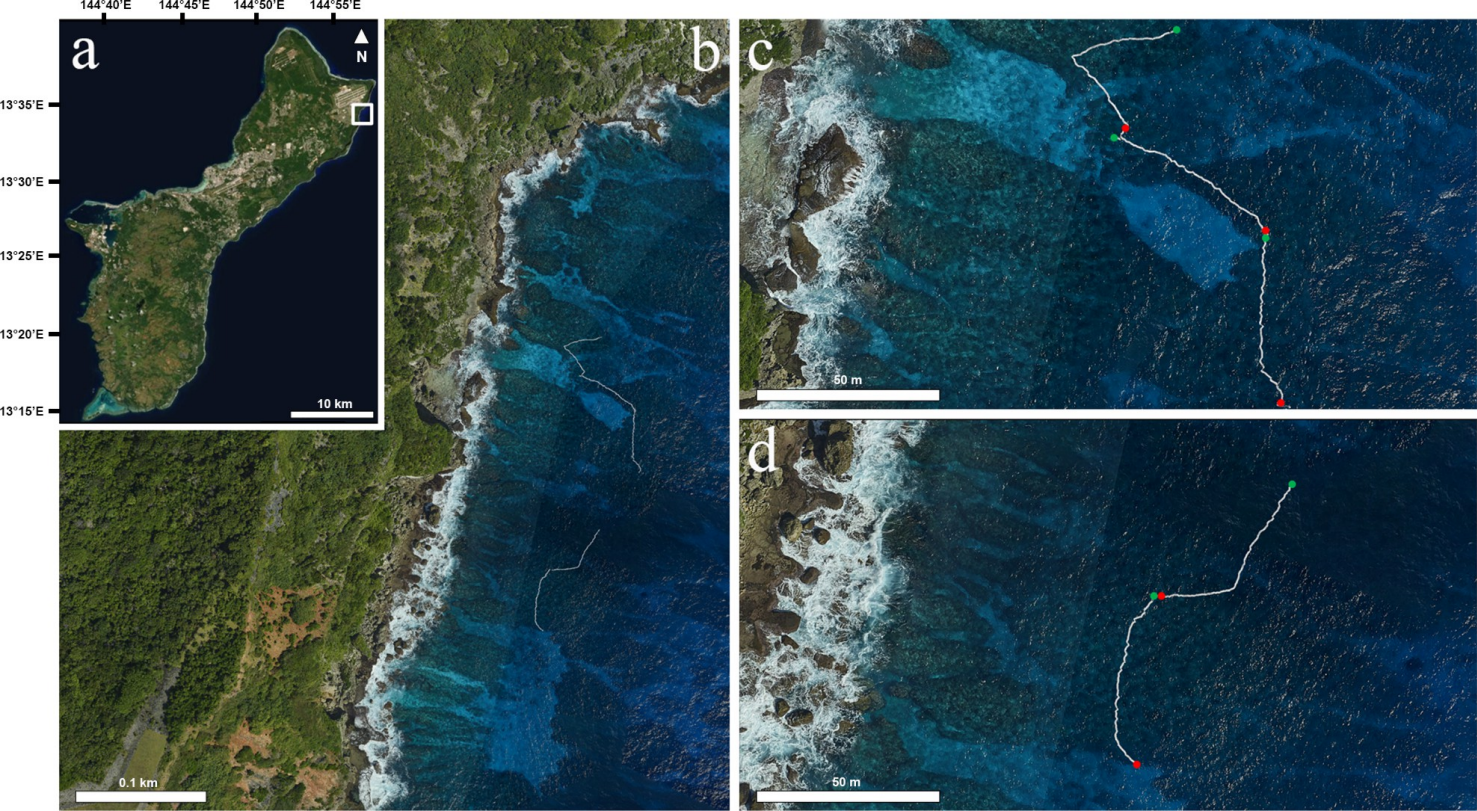
Maps depicting the study site. (a) Aerial map of Guam overlaid by a white box indicating the location of Lafac Bay. (b) Aerial map of the survey site showing the locations of the five transects surveyed (white lines). Transect maps were generated using the multiple navigation sensors of the DiveRay. (c,d) Maps enlarged to better visualize the survey area. The beginning and end of each transect are denoted by the green dots and red dots, respectively. Maps were created using ArcGIS Pro version 2.9 (Esri, Redlands, CA).

Benthic surveys were conducted at Lafac Bay, a coral reef site in the northeast of Guam (Fig 1). Access to the site is limited as it is situated next to a cliff line, just offshore from a military base, and far-removed from a boat ramp or marina. All surveys conducted were non-intrusive, non-destructive, and did not involve animal care or the collection of biological specimens. Therefore, permits or ethical approval were not required for this study. Access to reefs in northern and eastern Guam is seasonal because of intense wave action resulting from northeasterly trade winds in the dry season (January-June). This has resulted in a reduced exposure to anthropogenic stressors (e.g., overfishing, recreational/commercial use, and coastal development) that have adversely affected many of Guam’s reefs [82], making Lafac Bay one of the island’s most pristine nearshore reef ecosystems. Photoquadrat and hyperspectral surveys were conducted as described by Mills et al. [87] with minor modifications. The modified sampling protocols are summarized below.

### Photoquadrat survey methods

Five permanent 50 m transects at Lafac Bay were surveyed in 2015, 2017, and 2019 using a modified photoquadrat approach, providing a direct comparison of the benthic communities along these transects over a five-year period. Photos of 0.25 m^2^ quadrats were taken at every meter along each transect. Photos were cropped to the frame of the quadrat and post-processed to improve contrast and color. Each of the photos were then overlaid with 50 nonaligned, systematically sampled points, yielding 250 photos and 12,500 points of identification per survey. 58 benthic categories were used to identify most organisms to the highest taxonomic resolution possible (typically genus- or species-level) and included abiotic reef substrates (e.g., sediment, rubble, and bare substrate; S1 Table).

The 58 categories were aggregated into 10 broad taxonomic or functional benthic categories: *Acropora abrotanoides*, other Scleractinia, Corallinophycidae, Peyssonneliales, non-crustose red macroalgae, green macroalgae, brown macroalgae, Porifera, octocorals/hydrozoans, and turf algae/cyanobacteria/bare substrate. Broad benthic categories were selected due to their utility in rapid, macroscale reef monitoring and ecological/health assessments [88]. However, *Acropora abrotanoides* was placed in a separate category from other Scleractinia as it is a habitat-defining coral at the site. These categories were used to (1) examine pronounced changes in benthic community composition between survey years (S2 Table) and (2) evaluate diversity and evenness for each survey year using the Shannon Diversity Index (H) and Shannon Equitability Index (E), respectively [89].

### Hyperspectral survey methods

**Fig 2:**
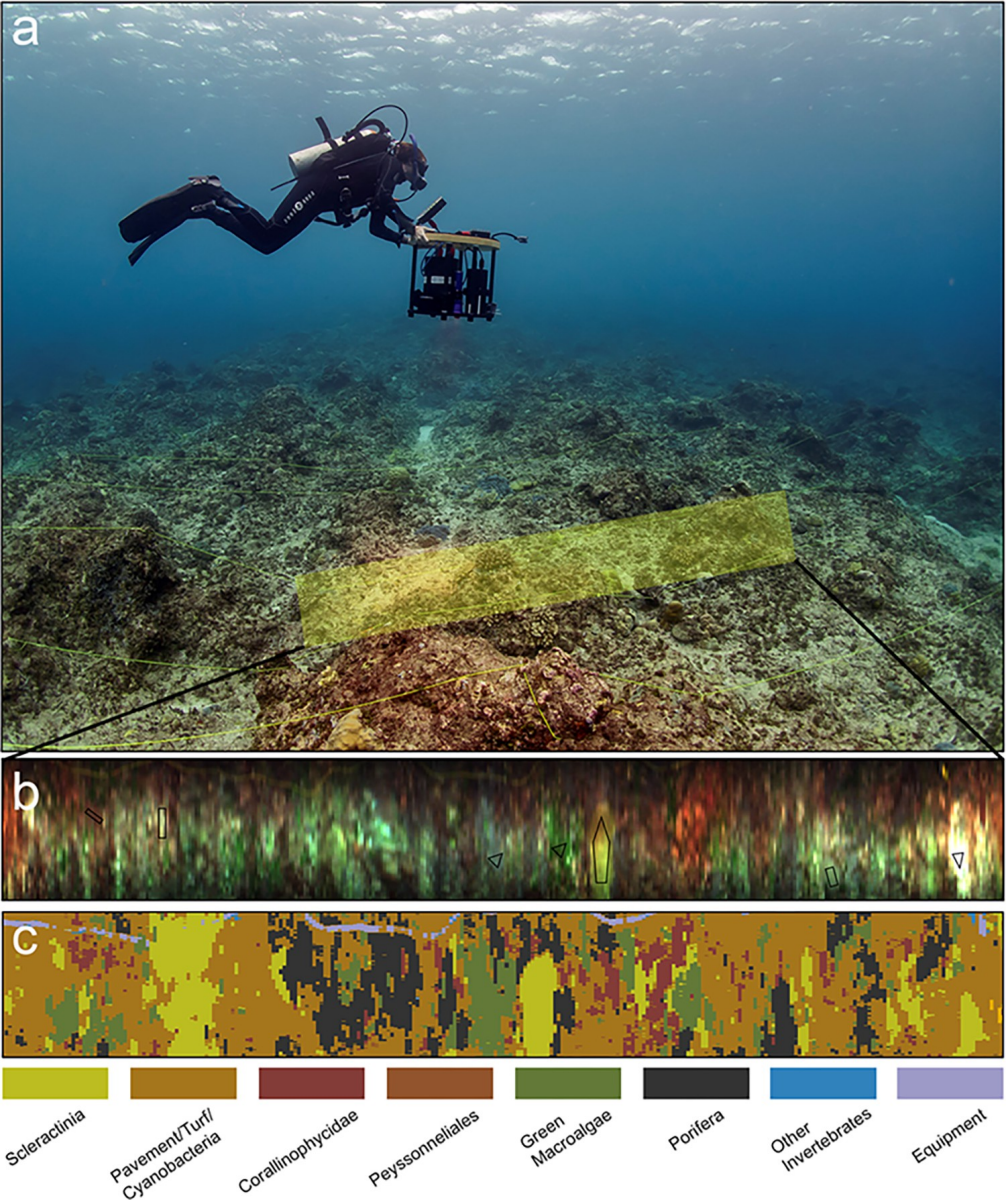
Protocol of reef surveys with the DiveRay. (a) Diver scans a swathe of reef (yellow rectangle) using the DiveRay. Note: this image was not taken at Lafac Bay. (b) Demarcation of regions of interest (ROIs; black polygons) in the hyperspectral scans, which are identified to the highest taxonomic resolution through verification of footage collected by the auxiliary RGB camera. The library of ROIs is used to automatically identify benthic categories through machine learning algorithms. (c) Georeferenced habitat map with a benthic category identification for each pixel in the scan.

Hyperspectral scans and photoquadrat images were taken concurrently in 2019. A beta version of the DiveRay was used to obtain the hyperspectral imagery (Fig 2; PlanBlue GmbH, Bremen, Germany). The DiveRay combines four principal components to record hyperspectral data: (1) a push-broom hyperspectral camera with a spectral resolution of roughly 2.9 nm (680 spatial pixels) and a field of view of approximately 30 degrees; (2) external full-spectrum lights (Keldan GmbH, Brügg, Switzerland) to collect hyperspectral data independent of lighting conditions; (3) multiple navigation sensors for georeferencing while scanning; and (4) a 5 MP low noise CMOS reference (RGB-)camera that records at a frame rate of 30 Hz to enable the annotation of hyperspectral data. For each transect recorded, a 10x10 cm matte grey reference plate was placed in the survey area to normalize the recorded spectra.

The DiveRay was used to scan 250 m^2^ of reef (5 transects x 50 m long x 1 m wide). Fused by an algorithm developed by PlanBlue, a combination of magnetic, inertial, and acoustic sensors were used to georeference each scan with a relative accuracy less than 0.5 m. Surveys were conducted approximately one meter above (and following the topography of) the reef to reduce scaling inaccuracies [74]. The hyperspectral camera is a push-broom or line-camera, so each frame records a line orthogonal to the swimming direction, with each pixel containing complete information within the visible and near-infrared spectra. In a first processing step, spectra were smoothed and standardized, and wavelengths without sufficient signal were removed. This pertains especially to the higher wavelengths, where water absorbs most of the signal. Spectra were then normalized using the reference plate to make the data comparable between transects.

User annotation effort was required before machine learning algorithms could be used to automatically classify the benthic communities. To do this, the HyperSuite computer software (PlanBlue GmbH) was used to discern benthic categories and annotate the hyperspectral scans. 450 regions of interest (ROIs), which each contained one of the 58 benthic categories, were delineated. These 450 ROIs covered roughly 120,000 pixels across all hyperspectral scans and were used as the library to train the machine learning algorithms prior to automated classification. To reduce observer bias, the benthic components present in all ROIs and photoquadrat points were identified by the same person. ROIs were then separated into the same functional groups as identified in the photoquadrat analyses. A proprietary deep learning based spectral-spatial Residual Neural Network (ResNet) model provided by PlanBlue was used for supervised classification [90]. The architecture and pre-activation concepts of the model are based on Zhong et al. [91] and He et al. [92], respectively. The model used the information from multiple spectra in a patch around a central pixel to create spectral and model-determined spatial features for each category. This was done by first reducing channel dimension via 1x1 convolutions, after which residual units [93] were created along the last dimension. The number of spectral features were then adjusted before spatial features could be created via residual units. The final classifier was created via average pooling over the window and softmax layer. The model was trained using 90% of the labeled pixels. This training process aimed to detect distinct differences between the different taxa or groups that are expected in ecological diversity surveys. The remaining 10% of labeled data were used as a validation set to assess how well the model performed on non-training data. The trained model was then used to predict a label for every recorded pixel in each transect, resulting in estimates of benthic community composition for each transect (S2 Table). The benthic cover estimates derived from the hyperspectral scans were then compared to those derived from the photoquadrat survey to assess if DiveRay data are a valid proxy for traditional benthic surveys.

### Statistical analyses

**Fig 3:**
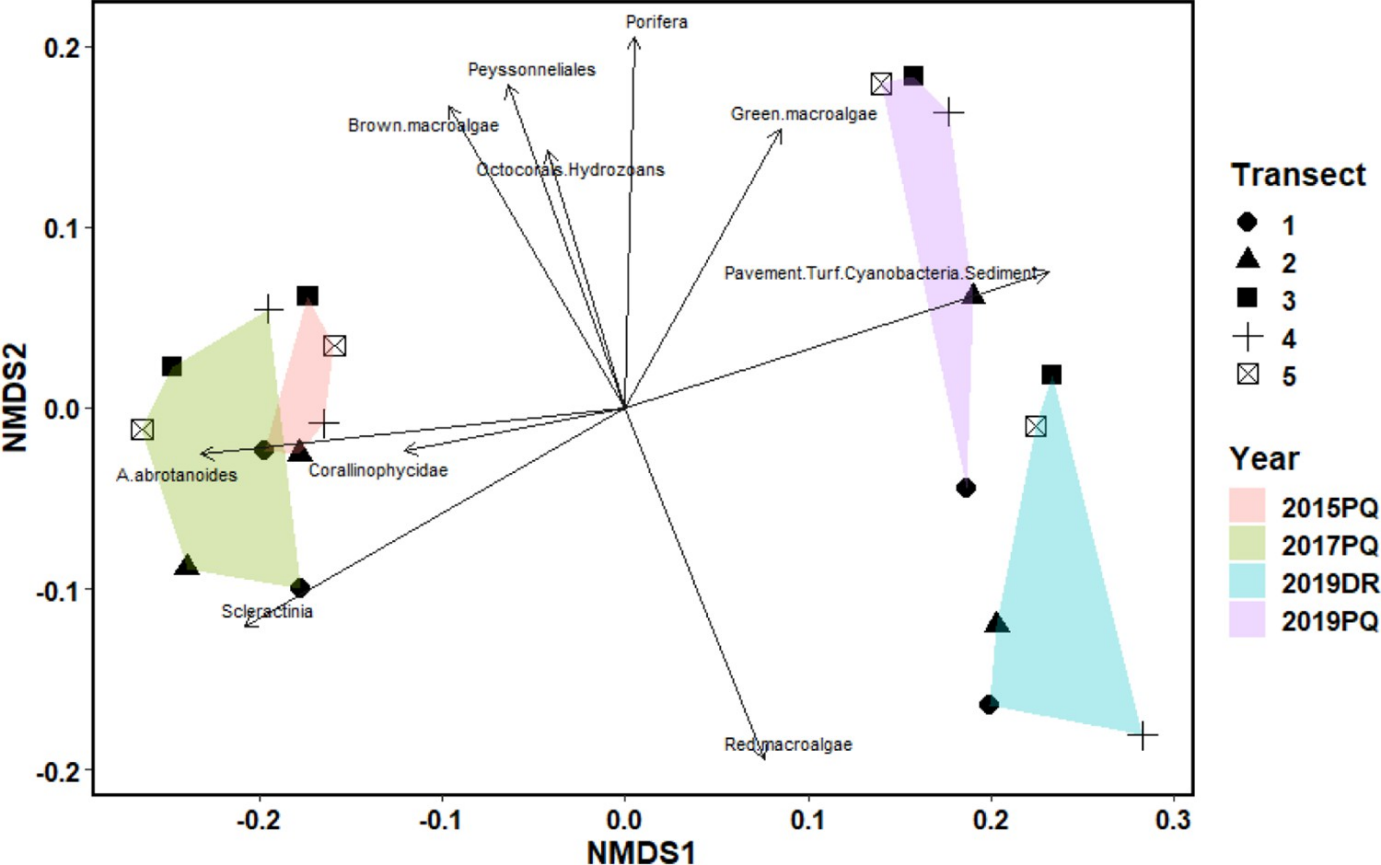
nMDS of benthic community composition with hulls overlaid for the photoquadrat (PQ) and DiveRay (DR) surveys. Vector arrows of benthic categories explain the differences in community composition between survey groups.

All statistical analyses were done using R version 4.1.3 (R Core Team, Vienna, Austria) and RStudio version 2022.02.1+461 (RStudio Team, Boston, MA, USA). Data were tested for normality using a Shapiro Wilk test prior to downstream analyses. Significance of temporal variations in the square root transformed cover of taxonomic or functional groups were tested using a one-way ANOVA and Tukey’s Honest Significant Difference test as a post hoc test. Differences in square root transformed community assemblages between surveys were visualized using a non-metric multidimensional scaling (nMDS) plot based on Bray-Curtis dissimilarity matrices and overlaid with hulls around each survey (Fig 3). An analysis of similarity (ANOSIM) test was conducted using the ‘vegan’ and ‘dplyr’ packages [94, 95] to determine if there are statistical differences between the four surveys. ANOSIM tests are non-parametric tests that use ranked dissimilarities to determine if differences between two or more groups (communities) are significant [96]. Since ANOSIM tests are non-parametric, untransformed benthic data was used to compare the communities estimated from each survey. These tests result in two outputs of interest: the ANOSIM statistic R and significance values. The former is a value between 0 and 1.0 where higher values denote higher dissimilarity. The latter value denotes whether the results are significant, where values less than 0.05 are considered statistically significant.

## Results

**Fig 4:**
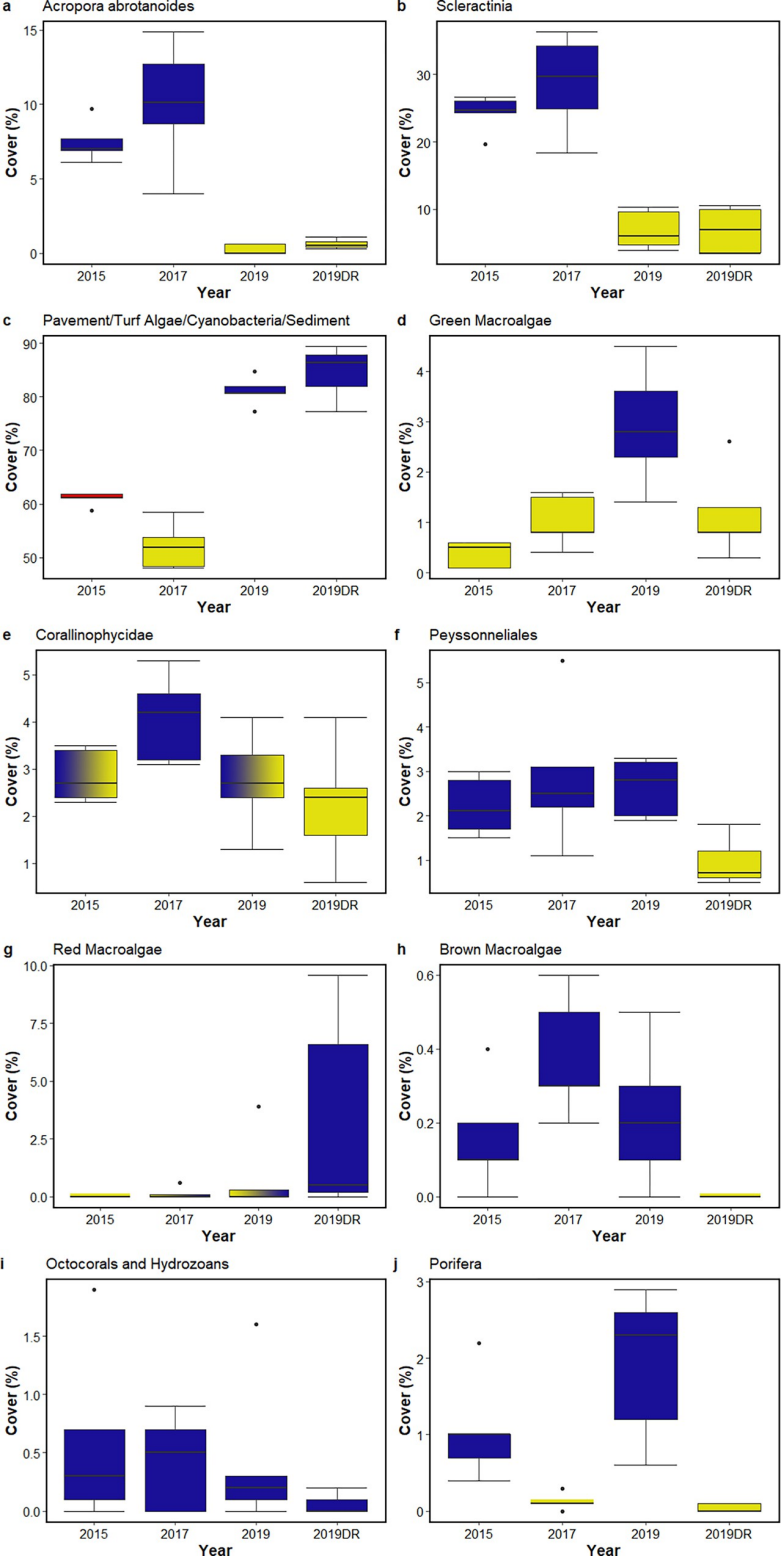
Box plots of percent cover of the benthic categories identified in the 2015, 2017, and 2019 photoquadrat surveys and the 2019 DiveRay survey. The DiveRay survey results are denoted by the ‘DR’ suffix. The percent cover in all four surveys is shown for (a) *Acropora abrotanoides*, (b) Scleractinia, (c) pavement, turf algae, cyanobacteria, sediment, (d) green macroalgae, (e) Corallinophycidae, (f) Peyssonneliales, (g) non-crustose red macroalgae, (h) brown macroalgae, (i) octocorals and hydrozoans, (j) Porifera. For each benthic category, cover differs significantly between surveys when boxplots do not contain the same color. In the plots, boxes show upper and lower quartiles, horizontal lines are the median values, whiskers denote the range of values, and dots represent outliers.

The average cover of turf algae, cyanobacteria, and bare substrate significantly decreased from 60.9 ± 1.1% in 2015 to 52.1 ± 3.8% in 2017 (±SD, n = 5 transects; Fig 4). In addition, mean cover of Porifera was significantly lower in 2017 (0.1 ± 0.1%) than in 2015 (1.1 ± 0.6%) (Fig 4). Mean cover of the other substrate categories were not statistically different between the two years (Table 1). Surveys in 2017 revealed an increase in diversity (H = 1.27) and evenness (E = 0.55) compared to 2015 (H = 1.14; E = 0.49). Community analyses revealed a significant difference in benthic community composition between 2015 and 2017 (ANOSIM statistic R = 0.564; significance = 0.017).

**Table 1:** Mean and standard deviations for each benthic category and for each survey year. Cover estimates for the year 2019 based on photoquadrats are denoted by the suffix "-PQ" and those based on hyperspectral data by the suffix "-DR". All values are rounded to the nearest tenth of a percent.

Category	2015	2017	2019-PQ	2019-DR
*Acropora abrotanoides*	7.5 ± 1.2%	10.1 ± 3.7%	0.2 ± 0.3%	0.6 ± 0.3%
Scleractinia	24.3 ± 2.5%	28.7 ± 6.5%	6.9 ± 2.6%	7.0 ± 2.7%
Green macroalgae	0.4 ± 0.2%	1.0 ± 0.4%	2.9 ± 1.1%	1.1 ± 0.8%
Turf algae, Cyanobacteria, Bare substrate	60.9 ± 1.1%	52.1 ± 3.8%	81.1 ± 2.4%	84.6 ± 4.5%
Corallinophycidae	2.9 ± 0.5%	4.0 ± 0.8%	2.8 ± 0.9%	2.3 ± 1.1%
Porifera	1.1 ± 0.6%	0.1 ± 0.1%	1.9 ± 0.9%	0.1 ± 0%
Peyssonneliales	2.2 ± 0.6%	2.9 ± 1.5%	2.6 ± 0.6%	1.0 ± 0.5%
Red macroalgae	0	0.1 ± 0.2%	0.9 ± 1.6%	3.4 ± 4.0%
Octocorals and Hydrozoans	0.6 ± 0.7%	0.4 ± 0.4%	0.4 ± 0.6%	0.1 ± 0.1%
Brown macroalgae	0.2 ± 0.1%	0.4 ± 0.2%	0.2 ± 0.2%	0

Surveys conducted in 2019 saw the near-complete disappearance of *A*. *abrotanoides* from Lafac Bay, with a decrease in mean cover from 10.1 ± 3.7% in 2017 to 0.2 ± 0.3% in 2019. The cover of other hard corals also decreased substantially during this time frame, from 28.7 ± 6.5% in 2017 to 6.9 ± 2.6% in 2019. These losses in hard coral cover coincided with a significant increase in the combined cover of turf algae, cyanobacteria, and bare substrate, rising from 52.1 ± 3.8% in 2017 to 81.1 ± 2.4% in 2019. Minor increases in cover were documented for sponges (from 0.1 ± 0.1% in 2017 to 1.9 ± 0.9% in 2019) and green macroalgae (particularly *Halimeda* species; from 1.0 ± 0.4% to 2.9 ± 1.1%). No significant change in the mean benthic cover of other substrate categories was observed between 2017 and 2019 (Table 1). The pronounced change in cover for certain benthic categories was reflected in a significant difference in overall community composition between 2017 and 2019 (ANOSIM statistic R = 1; significance = 0.009), as well as among all three survey years (ANOSIM statistic R = 0.884; significance = 0.0001). This significant difference in community composition was also mirrored by a reduction in diversity (H = 0.82) and evenness (E = 0.36) in 2019, when compared to 2017 (H = 1.27; E = 0.55).

In general, the machine learning model accurately predicted the 10% of labeled data set aside as a validation set. With the exception of red macroalgae, the proportion of predicted labels that matched the true labels in each benthic category ranged from 0.88 (Peyssonneliales) to 1.0 (*A*. *abrotanoides* and brown macroalgae). However, the proportion of predicted and true labels for red macroalgae was 0.52, with 34% of labels predicted to be turf algae/cyanobacteria/bare substrate. Automatically classified benthic cover estimates derived from the hyperspectral scans in 2019 were largely similar to those derived from photoquadrat surveys (Table 1), particularly those of the most dominant benthic categories. Cover estimates of *A*. *abrotanoides*, other hard corals, turf algae/cyanobacteria/bare substrate, Corallinophycidae, octocorals/hydrozoans, red macroalgae, and other invertebrates were determined not to be statistically different between the two survey techniques. Significant differences in cover estimates were found for four substrate categories (green macroalgae, Porifera, Peyssonneliales, and brown macroalgae), which made a minor contribution to total benthic cover. Community analyses, however, revealed no significant difference between the estimates derived from the hyperspectral and photoquadrat surveys (ANOSIM statistic R = 0.284; significance = 0.062).

## Discussion

### Benthic community shift

**Fig 5:**
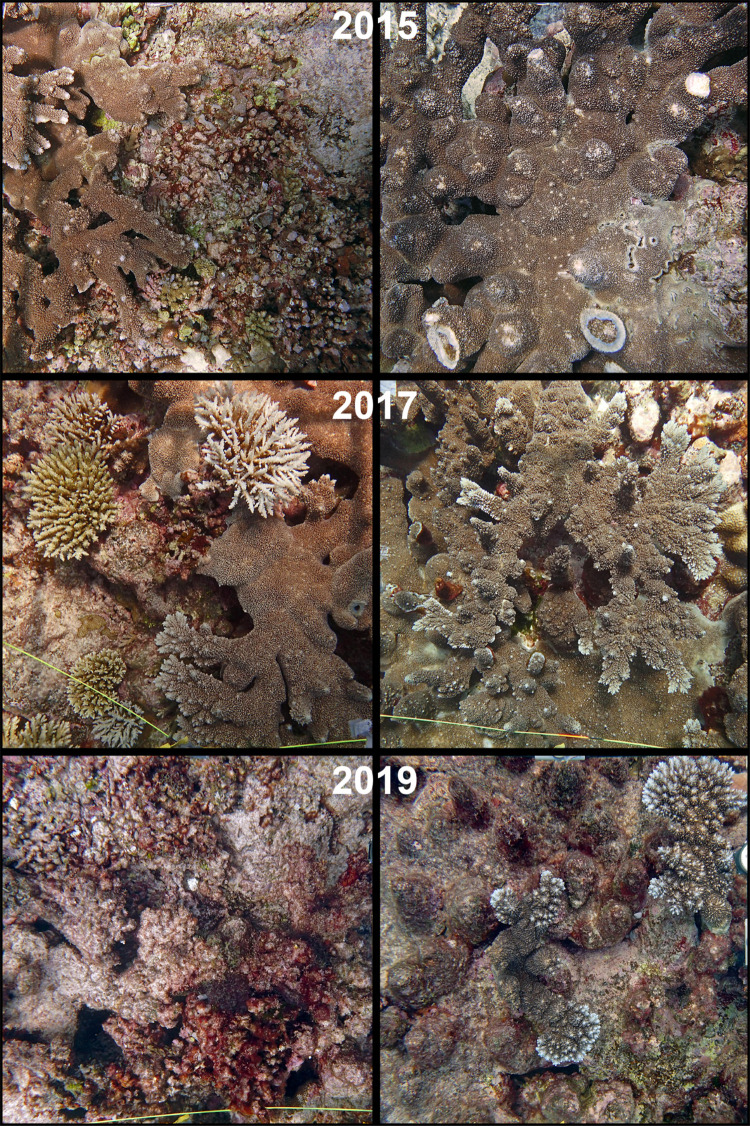
Images demonstrating the shift in benthic communities in 2015 (top row), 2017 (middle row), and 2019 (bottom row). Photoquadrats in the first column are from the midpoint in the first transect, those in the second column are from the midpoint in the third transect.

This study documents a rapid degradation of a healthy and diverse coral reef community within the time span of two years and demonstrates the value of underwater hyperspectral imaging for reef monitoring. Despite the occurrence of a severe bleaching event and extreme low tides between surveys in 2015 and 2017 [84], and contrary to the decline in scleractinian cover observed elsewhere in Guam [83], the average cover of *A*. *abrotanoides* and other hard corals at Lafac Bay was not affected (Fig 5). However, many more juvenile corals were identified in 2015 (n = 48) than in 2017 (n = 14), all but one of which were fast-growing but fragile branching or digitate *Acropora* species. Excluding the staghorn *A*. *abrotanoides*, which was in its own category, 34.8% (47 out of 135) of all points identified as other *Acropora* corals in 2015 were identified as juvenile. In addition to the island-wide bleaching and low tide events in 2013 and 2014, as well as the ENSO-triggered extreme low tides in 2014 and 2015 [84], the 2015 surveys were conducted shortly after typhoon Dolphin passed north of Guam, which caused some of the fragile *Acropora* species to break or fragment (Fig 5, top right image). Despite this, the reef community at Lafac Bay appears to have been resilient or capable of recovery through successful coral recruitment prior to the surveys in 2015. This could, at least in part, be due to the high degrees of wave action, water circulation, and flushing at the reef, which has been observed to mitigate the effects of extreme low tides and warm temperature periods to an extent [83]. By comparison, despite having over double the number of points identified as other *Acropora* corals, only 4.4% (14 out of 319) of these points were identified as juvenile in 2017.

Moreover, while the cover of *A*. *abrotanoides* and other Scleractinia did not significantly differ, the cover of all hard corals combined was significantly higher (p = 0.01) in 2017 (38.8 ± 4.3%) than in 2015 (31.8 ± 1.9%). Since corals often compete with turf algae for space on the reef [97], this could also explain the significant decrease in turf algae, cyanobacteria, and bare substrate cover. The significant increase in total combined coral cover, the stability of reef building and cementing coralline algae (Corallinophycidae; Fig 4E), the significant decrease in turf algae, cyanobacteria, and bare substrate (Fig 4C), and the increase in community diversity between 2015 and 2017 provide further support for the resiliency of Lafac’s reef communities. Although the differences in scleractinian coral cover between 2015 and 2017 were not as pronounced as the decline between 2017 to 2019, the significant difference in community composition between 2015 and 2017 suggests that the reefs in 2015 were bouncing back from environmental impacts in the previous years [98, 99].

In 2017, the most severe and damaging coral bleaching event recorded for Guam resulted in a substantial loss in scleractinian coral cover island-wide [84]. Lafac Bay also saw a sharp decline in coral cover and benthic community diversity between 2017 and 2019. One of the most prominent changes was the almost complete mortality of the staghorn coral *Acropora abrotanoides*, which decreased in average cover from 10.1 ± 3.7% in 2017 to 0.2 ± 0.3% in 2019 (Fig 4A). *A*. *abrotanoides* is an ecosystem engineer that shapes reefs and provides habitats to a suite of organisms, making its mass disappearance in 2019 all the more striking (Fig 5). Other scleractinian corals shared a similar fate as *A*. *abrotanoides*, suffering a significant decline in average coral cover from 28.7 ± 6.5% in 2017 to 6.9 ± 2.6% in 2019 (Fig 4B). The observed mass mortality of *A*. *abrotanoides* and other scleractinian corals at Lafac Bay is consistent with similar declines around Guam [83, 84, 86] and globally [100–104]. While not statistically significant, the average cover of Corallinophycidae decreased to 2.3 ± 1.1%, an amount similar to what was observed in 2015. These algae serve important ecological functions like reef building, cementing, suppression of nutrient indicator algae, and the promotion of invertebrate larval settlement [105–109]. The decline in coral and Corallinophycidae cover coincided with a near 50% increase in turf algae/cyanobacteria/bare substrate (from 52.1 ± 3.8% in 2017 to 81.1 ± 2.4% in 2019). Turf algae and cyanobacteria were observed overgrowing the dead skeletons of *A*. *abrotanoides* and other coral colonies across all transects. Proportionally large increases in the average cover of Porifera and green macroalgae, *Halimeda* species in particular, were also observed. Interestingly, the average cover of encrusting red algae of the order Peyssonneliales, some of which are known to outcompete and overgrow corals and other invertebrates on disturbed reefs [35, 110–113], remained largely constant throughout the three surveys. Prior to the disturbance events in 2013, hard corals determined benthic cover at Lafac Bay [82, 114]. While reefs with high coral and coralline algal cover (‘foundation species’) resist the propagation of turf algae and cyanobacteria, increased competition following disturbance events can inhibit reef recovery and allow turf algae to proliferate [97]. Once established, turf algae can severely affect coral growth and tissue mortality [97]. This can compound with the increase in turf algae/cyanobacteria and help lead to the shift in competitive balance between foundation species and turf algae/cyanobacteria. The loss of coral cover in conjunction with a significant increase in turf algae, cyanobacteria, and other macroalgae could indicate that the reefs at Lafac Bay are in the process of shifting from a coral to an algal-dominated state [33]. The pronounced shift in benthic community composition is also shown in Fig 3, where the nMDS plot provides a visual representation of the benthic communities observed for each survey as well as the benthic component(s) that contributed most to the variation in community composition. *A*. *abrotanoides*, other scleractinian corals, and Corallinophycidae best described the community composition in 2015 and 2017. Whereas in 2019, the benthic communities were characterized by an increased cover in turf algae/cyanobacteria/bare substrate, red macroalgae, and green macroalgae.

The severe coral bleaching event in 2017 was, in no small part, a result of the highest accumulated heat stress recorded since satellite measurements began [84]. Extensive coral mortality and increases in turf algae, cyanobacteria, and bare substrate in 2019 were a result of the combined pressure of unprecedented heat stress and the cumulative effects of the bleaching and low tide events in 2013, 2014, 2016, and 2017, as well as the ENSO-triggered extreme low tides in 2014 and 2015 [84]. Despite the significant decline in live coral cover, the structural complexity of reefs at Lafac Bay remained high (average rugosity of 2.81 and slope of 44 degrees) [115], indicating that the reef had not ‘flattened’ or eroded yet [28]. Examples of coral recovery following acute or recurrent disturbances have been reported for the tropical Pacific [37, 99]. Reefs like those at Lafac Bay, which experience limited anthropogenic impacts and are primarily disturbed by natural events, have been observed to return to a coral-dominated state following a stage of macroalgal dominance [116]. The capacity to recover following severe disturbances, paired with the reversal of macroalgal-dominant phase shifts observed on similar reefs, make Lafac Bay an excellent candidate to monitor the succession of reef communities following natural disturbance events. Future monitoring should not only focus on corals but also include less obvious taxa (e.g., octocorals, crustose calcifying red algae, other macroalgae, sponges, hydrozoans, etc.) in an effort to document changes at the community level.

### Underwater hyperspectral Imaging

The estimated cover of seven of the eleven benthic categories did not differ significantly between the two survey methods, including *A*. *abrotanoides*, other Scleractinia, Corallinophycidae, and turf algae/cyanobacteria/bare substrate. All these taxa were well-represented in the annotation libraries. However, the model that predicted the benthic category for each pixel of the hyperspectral scans did not perform as well when recognizing small, infrequent organisms or heterogenous patches. The four substrate categories of which cover estimates differed between the photoquadrat and DiveRay surveys were: green macroalgae, Porifera, Peyssonneliales, and brown macroalgae. The contribution of these four categories to overall community composition was minor. The taxa in these categories were often small, cryptic, infrequent (all of them had less than 3% average cover), and occurred in heterogeneous patches. Therefore, despite a pixel resolution smaller than one centimeter, these organisms were difficult to detect by the hyperspectral imager and were not well-represented in the annotation libraries.

This also contributed to why communities were not predicted at higher taxonomic resolutions, as the low cover of everything other than turf algae, cyanobacteria, and bare substrate resulted in the under-representation of many taxa (e.g., *Montipora* spp., *Lobophora* spp., *Cyphastrea* spp., etc.) in the annotation libraries. The ROIs identified in this study were added to a library that had already been established for Guam [87] since annotation libraries can be exchanged between survey efforts of similar reef environments [117]. However, while the previously established library helped the model identify some of the more frequently-occurring taxa (such as *Porites* spp. or *Leptoria phrygia*), the existing library may not have been sufficient to account for the high biodiversity of Guam’s reefs [87]. The identification accuracy of these groups, as well as the identification of taxa at higher taxonomic resolutions, will likely improve as increased sampling and annotation efforts produce more robust annotation libraries. Despite significant differences in the cover estimates of four substrate categories, the community analyses showed no significant difference between the two survey methods (Figs 3 and 4). Similar to what was concluded by Mills et al. [87], these results suggest that this methodology can accurately estimate benthic community composition using broad taxonomic or functional group categories. This could prove useful for applications such as rapid ecological assessments, long-term (repeated) benthic monitoring, or monitoring live coral cover, a primary indicator used to investigate the global decline in reef health [88].

Diver-operated hyperspectral imaging and automated image classification could expedite benthic surveys. The photoquadrat method is time-consuming and labor intensive, which is not ideal for monitoring projects that require frequent surveys of large areas. Surveying large stretches of reef is important to properly document the patchiness and clustering of organisms and habitats [118, 119]. Hyperspectral analysis could potentially address these issues and produce accurate, rapid results. Site selection is an important consideration when conducting underwater hyperspectral surveys, as estimates of benthic cover and community composition have been confounded on reefs of low macrobenthic species richness, with high structurally complexity, and situated along steep slopes [87]. However, despite the significant decline in coral cover, Lafac Bay is still structurally complex [115]. As such, underwater hyperspectral imaging is capable of surveying reefs with considerable terrain complexity.

The DiveRay is easy to operate underwater and data collection is fast as scans are completed during a casual swim along a transect. Whereas photoquadrat surveys took up to an hour to complete, the hyperspectral surveys in this study were completed in roughly 30 minutes. Repeat photoquadrat and hyperspectral surveys also require transect lines to be deployed and recovered. However, the underwater navigation sensors of the DiveRay allow hyperspectral scans to be georeferenced while surveying, which could facilitate rapid monitoring of large reef areas and produce georeferenced maps (Fig 1B–1D). The creation of an annotation library requires a significant investment of time and effort. In this study, the annotation of hyperspectral scans took less than one week, while photoquadrat annotation analysis for one survey year took 2–4 weeks.

Once robust libraries of ROIs are produced, subsequent hyperspectral processing is automated and can be completed in a fraction of the time (minutes to hours). The time investment for manual photoquadrat annotations stays at a constant rate per identification point. Past hyperspectral scans can also be re-analyzed as libraries are updated and improved over time. So, while the initial expenditure to conduct hyperspectral surveys seems higher than those of photoquadrat surveys, repetitive monitoring efforts of large areas see cost savings in subsequent surveys [49, 74]. Considering the need for increased reef monitoring at local and regional scales [41, 120, 121], libraries built on surveys from different habitats and with good geographical coverage could serve large-scale monitoring efforts.

The reefs at Lafac Bay have demonstrated the ability to recover following disturbance events that were catastrophic for other reefs around Guam. After the 2017 bleaching event, however, Lafac Bay suffered a significant loss in coral cover and the almost total demise of the ecosystem engineer *Acropora abrotanoides*. Reefs that experience limited anthropogenic impacts can recover from severe disturbances and return to a coral-dominated state [116]. Lafac Bay is an excellent candidate to evaluate the recovery potential of western Pacific reefs following natural disturbance events. Underwater hyperspectral imaging and automated benthic classification remain novel approaches to reef monitoring [74, 87, 117, 122–124], which can provide accurate estimates of benthic community composition. These advancing technologies will benefit monitoring programs, especially those that rely on frequent, rapid, and accurate assessments of dynamic reef communities like those at Lafac Bay.

## Supporting information

S1 Table List of categories used to identify taxa and abiotic reef substrates in photoquadrats and hyperspectral scans. The second column lists the broad taxonomic or functional group each ID category was placed in for further analysis.
(XLSX)

S2 Table Estimated cover (%) of the benthic categories identified in the 2015, 2017, and 2019 surveys. Cover estimates for the year 2019 based on photoquadrats are denoted by the suffix "-PQ" and those based on hyperspectral data by the suffix "-DR".
(CSV)

## References

[1] Reaka-Kudla ML. The global biodiversity of coral reefs: A comparison with rain forests. In: Reaka-Kudla ML, Wilson DE, Wilson EO, editors. Biodiversity II. Washington, DC: Joseph Henry Press; 1997. pp 83–107.

[2] Birkeland C. Life and death of coral reefs. New York: Chapman and Hall; 1997.

[3] Sing Wong A, Vrontos S, Taylor ML. An assessment of people living by coral reefs over space and time. Glob Change Biol. 2022; 28: 7139–7153. doi: 10.1111/gcb.16391 36168958 PMC9827914

[4] Burns JHR, Delparte D, Kapono L, Belt M, Gates RD, Takabayashi M. Assessing the impact of acute disturbances on the structure and composition of a coral community using innovative 3D reconstruction techniques. Methods Oceanogr. 2016; 15–16: 49–59.

[5] Kanki T, Nakamoto K, Hayakawa J, Kitagawa T, Kawamura T. A new method for investigating relationships between distribution of sessile organisms and multiple terrain variables by photogrammetry of subtidal bedrocks. Front Mar Sci. 2021; 8: 654950. doi: 10.3389/fmars.2021.654950

[6] Bozec YM, Mumby P. Synergistic impacts of global warming on the resilience of coral reefs. Philos Trans R Soc Lond B Biol Sci. 2015; 370: 20130627. doi: 10.1098/rstb.2013.0267

[7] Pandolfi JM, Bradbury RH, Sala E, Hughes TP, Bjorndal KA, Cooke RG, et al. Global trajectories of the long-term decline of coral reef ecosystems. Science. 2003; 301: 955–958. doi: 10.1126/science.1085706 12920296

[8] D’agata S, Mouillot D, Kulbicki M, Andréfouët S, Bellwood DR, Cinner JE, et al. Human-mediated loss of phylogenetic and functional diversity in coral reef fishes. Curr Biol. 2014; 24: 555–560. doi: 10.1016/j.cub.2014.01.049 24560574

[9] Hughes TP, Barnes ML, Bellwood DR, Cinner JE, Cumming GS, Jackson JBC et al. Coral reefs in the Anthropocene. Nature. 2017; 546: 82–90. doi: 10.1038/nature22901 28569801

[10] Hoegh-Guldberg O. Climate change, coral bleaching and the future of the world’s coral reefs. Mar Freshw Res. 1999; 50: 839–866. doi: 10.1071/MF99078

[11] Hoegh-Guldberg O, Mumby PJ, Hooten A, Steneck RS, Greenfield P, Gomez E et al. Coral reefs under rapid climate change and ocean acidification. Science. 2007; 318: 1737–1742. doi: 10.1126/science.1152509 18079392

[12] Hughes TP, Kerry JT, Álvarez-Noriega M, Álvarez-Romero JG, Anderson KD, Baird AH et al. Global warming and recurrent mass bleaching of corals. Nature. 2017; 543: 373–377. doi: 10.1038/nature21707 28300113

[13] Eyre BD, Cyronak T, Drupp P, De Carlo EH, Sachs JP, Andersson AJ. Coral reefs will transition to net dissolving before end of century. Science. 2018; 359, 908–911. doi: 10.1126/science.aao1118 29472482

[14] Hughes TP, Kerry JT, Baird AH, Connolly SR, Dietzel A, Mark Eakin C et al. Global warming transforms coral reef assemblages. Nature. 2018; 556: 492–496. doi: 10.1038/s41586-018-0041-2 29670282

[15] Perry CT, Alvarez-Filip L, Graham NAJ, Mumby PJ, Wilson SK, Kench PS et al. Loss of coral reef growth capacity to track future increases in sea level. Nature. 2018; 558: 396–400. doi: 10.1038/s41586-018-0194-z 29904103

[16] Knowlton N. The future of coral reefs. Proc Natl Sci USA. 2001; 98: 5419–5425. doi: 10.1073/pnas.091092998 11344288 PMC33228

[17] Williams GJ, Graham NAJ. Rethinking coral reef functional futures. Funct Ecol. 2019; 33: 942–947. doi: 10.1111/1365-2435.13374

[18] Graham NAJ, Nash KL. The importance of structural complexity in coral reef ecosystems. Coral Reefs. 2013; 32: 315–326. doi: 10.1007/s00338-012-0984-y

[19] Wangpraseurt D, Pernice M, Guagliardo P, Kilburn MR, Clode PL, Polerecky L, et al. Light microenvironment and single-cell gradients of carbon fixation in tissues of symbiont-bearing corals. ISME J. 2015; 10: 788–792. doi: 10.1038/ismej.2015.133 26241503 PMC4817679

[20] D’Elia CF. The cycling on essential elements in coral reefs. In: Pomeroy LR, Alberts JJ, editors. Concepts of Ecosystem Ecology. New York: Springer-Verlag; 1988. pp. 195–230.

[21] Davies PJ, Hutchings PA. Initial colonization, erosion and accretion of coral substrate. Coral Reefs. 1983; 2: 27–35.

[22] Masselink G, Beetham E, Kench P. Coral reef islands can accrete vertically in response to sea level rise. Sci Adv. 2020; 6: eaay3656. doi: 10.1126/sciadv.aay3656 32577502 PMC7286686

[23] Bellwood DR, Pratchett MS, Morrison TH, Gurney GG, Hughes TP, Álvarez-Romero JG, et al. Coral reef conservation in the Anthropocene: Confronting spatial mismatches and prioritizing functions. Biol Conserv. 2019; 236: 604–615. doi: 10.1016/j.biocon.2019.05.056

[24] Ford HV, Gove JM, Healey JR, Davies AJ, Graham NAJ, Williams GJ. Recurring bleaching events disrupt the spatial properties of coral reef benthic communities across scales. Remote Sens Ecol Conserv. 2023; doi: 10.1002/rse2.355

[25] Pratchett MS, Thompson CA, Hoey AS, Cowman PF, Wilson SK. Effects of coral bleaching and coral loss on the structure and function of reef fish assemblages. In: van Oppen MJH, Lough JM, editors. Coral bleaching: Patterns, processes, causes and consequences. Switzerland: Springer; 2018. pp. 265–293.

[26] Eddy TD, Lam VWY, Reygondeau G, Cisneros-Montemayor AM, Greer K, Palomares MLD, et al. Global decline in capacity of coral reefs to provide ecosystem services. One Earth. 2021; 4: 1278–1285. doi: 10.1016/j.oneear.2021.08.016

[27] Rädecker N, Pogoreutz C, Gegner HM, Cárdenas A, Roth F, Bougoure J, et al. Heat stress destabilizes symbiotic nutrient cycling in corals. Proc Natl Acad Sci USA. 2021; 118: e2022653118. doi: 10.1073/pnas.2022653118 33500354 PMC7865147

[28] Álvarez-Filip L, Dulvy NK, Gill JA, Côté IM, Watkinson AR. Flattening of Caribbean coral reefs: Region-wide declines in architectural complexity. Proc R Soc Biol Sci. 2009; 276: 3019–3025. doi: 10.1098/rspb.2009.0339 19515663 PMC2817220

[29] Molina-Hernández A, Medellín-Maldonado F, Lange ID, Perry CT, Álvarez-Filip L. Coral reef erosion: In situ measurement on different dead coral substrates on a Caribbean reef. Limnol Oceanogr. 2022; 67: 2734–2749. doi: 10.1002/lno.12234

[30] Pratchett MS, Heron SF, Mellin C, Cumming GS. Recurrent mass-bleaching and the potential for ecosystem collapse on Australia’s Great Barrier Reef. In: Canadell JG, Jackson RB, editors. Ecosystem Collapse and Climate Change. Switzerland: Springer; 2021. pp. 265–289.

[31] Done TJ. Phase shifts in coral reef communities and their ecological significance. Hydrobiologia. 1992; 247: 121–132. doi: 10.1007/BF00008211

[32] Knowlton N. Thresholds and multiple stable states in coral reef community dynamics. Am Zool. 1992; 32: 674–682. doi: 10.1093/icb/32.6.674

[33] Bellwood DR, Hughes TP, Folke C, Nyström M. Confronting the coral reef crisis. Nature. 2004; 429: 827–833. doi: 10.1038/nature02691 15215854

[34] Schils T. Branching *Lithophyllum* coralline algae: dominant reef builders on herbivory-depressed tropical reefs after high coral mortality. Diversity. 2023; 15: 1025. doi: 10.3390/d15091025

[35] Edmunds PJ, Schils T, Wilson B. The rising threat of peyssonnelioid algal crusts on coral reefs. Curr Biol. 2023; 33: R1140–R1141. doi: 10.1016/j.cub.2023.08.097 37935123

[36] Bell JJ. The functional roles of marine sponges. Estuar Coast Shelf Sci. 2008; 79: 341–353. doi: 10.1016/j.ecss.2008.05.002

[37] Schils T. Episodic eruptions of volcanic ash trigger a reversible cascade of nuisance species outbreaks in pristine coral habitats. PLoS ONE. 2012; 7: e46639. doi: 10.1371/journal.pone.0046639 23056381 PMC3464252

[38] Work TM, Aeby GS, Neal BP, Price NN, Conklin E, Pollock A. Managing an invasive corallimorph at Palmyra Atoll National Wildlife refuge, Line Islands, Central Pacific. Biol Invasions. 2018; 20: 2197–2208. doi: 10.1007/s10530-018-1696-1

[39] Reimer JD, Wee HB, López C, Beger M, Cruz ICS. Widespread Zoanthus and Palythoa dominance, barrens, and phase shifts in shallow water subtropical and tropical marine ecosystems. Oceanogr Mar Biol. 2021; 59: 533–558. doi: 10.1201/9781003138846-7

[40] Soares MO, Kitahara MV, Santos MEA, Bejarano S, Rabelo EF, Cruz ICS. The flourishing and vulnerabilities of zoantharians on Southwestern Atlantic reefs. Mar Environ Res. 2022; 173: 105535. doi: 10.1016/j.marenvres.2021.105535 34879290

[41] Dietzel A, Bode M, Connolly SR, Hughes TP. The population sizes and global extinction risk of reef-building coral species at biogeographic scales. Nat Ecol Evol. 2021; 5: 663–669. doi: 10.1038/s41559-021-01393-4 33649542

[42] Saunders MI, Leon JX, Callaghan DP, Roelfsema CM, Hamylton S, Brown CJ, et al. Interdependency of tropical marine ecosystems in response to climate change. Nat Clim Change. 2014; 4: 724–729. doi: 10.1038/nclimate2274

[43] Grigg RW, Maragos JE. Recolonization of hermatypic corals on submerged lava flows in Hawaii. Ecology. 1974; 55: 387–395. doi: 10.2307/1935226

[44] Roelfsema C, Kovacs EM, Vercelloni J, Markey K, Rodriguez-Ramirez A, Lopez-Marcano S, et al. Fine-scale time series surveys reveal new insights into spatio-temporal trends in coral cover (2002–2018), of a coral reef on the Southern Great Barrier Reef. Coral Reefs. 2021; 40: 1055–1067. doi: 10.1007/s00338-021-02104-y

[45] Foster MS, Harrold C, Hardin DD. Point vs. photo quadrat estimates of the cover of sessile marine organisms. J Exp Mar Biol Ecol. 1991; 146: 193–203. doi: 10.1016/0022-0981(91)90025-R

[46] Lechene MAA, Haberstroh AJ, Byrne M, Figueira W, Ferrari R. Optimising sampling strategies in coral reefs using large-area mosaics. Remote Sens. 2019; 11: 2907. doi: 10.3390/rs11242907

[47] Ahmad W, Neil DT. An evaluation of Landsat Thematic Mapper ™ digital data for discriminating coral reef zonation: Heron Reef (GBR). Int J Remote Sens. 1994; 15: 2583–2597. doi: 10.1080/01431169408954268

[48] Andréfouët S, Kramer P, Torres-Pulliza D, Joyce KE, Hochberg EJ, Garza-Perez R, et al. Multi-site evaluation of IKONOS data for classification of tropical coral reef environments. Remote Sens Environ. 2003; 88: 128–143. doi: 10.1016/j.rse.2003.04.005

[49] Roelfsema C, Phinn S. Integrating field data with high spatial resolution multispectral satellite imagery for calibration and validation of coral reef benthic community maps. J Appl Remote Sens. 2010; 4: 043527. doi: 10.1117/1.3430107

[50] Goodman JA, Purkis SJ, Phinn SR, editors. Coral reef remote sensing: A guide for mapping, monitoring, and management. 1st ed. Dordrecht: Springer Dordrecht; 2013.

[51] Choi JK, Eom J, Moon JE, Kim K, Cho S. Monitoring coral reef habitat changes using high-spatial-resolution satellite images. In: Kim SW, Choi YU, Kim T, Park HS, editors. Coral reefs of Chuuk, Federated States of Micronesia. Chuuk State: Korea South Pacific Ocean Research Center (KSORC); 2014. pp. 21–25.

[52] Liu G, Heron SF, Eakin CM, Muller-Karger FE, Vega-Rodriguez M, Guild LS, et al. Reef-scale thermal stress monitoring of coral ecosystems: New 5-km global products from NOAA Coral Reef Watch. Remote Sens. 2014; 6: 11579–11606. doi: 10.3390/rs61111579

[53] Castellanos-Galindo GA, Casella E, Mejía-Rentería JC, Rovere A. Habitat mapping of remote coasts: Evaluating the usefulness of lightweight unmanned aerial vehicles for conservation and monitoring. Biol Conserv. 2019; 239: 108282. doi: 10.1016/j.biocon.2019.108282

[54] Roelfsema CM, Kovacs EM, Ortiz JC, Callaghan DP, Hock K, Mongin M, et al. Habitat maps to enhance monitoring and management of the Great Barrier Reef. Coral Reefs. 2020; 39: 1039–1054. doi: 10.1007/s00338-020-01929-3

[55] Chirayath V, Instrella R. Fluid lensing and machine learning for centimeter-resolution airborne assessment of coral reefs in American Samoa. Remote Sens Environ. 2019; 235: 111475. doi: 10.1016/j.rse.2019.111475

[56] Olden JD, Lawler JL, LeRoy Poff N. Machine learning methods without tears: a primer for ecologists. Q Rev Biol. 2008; 83: 171–193. doi: 10.1086/587826 18605534

[57] Peters DPC, Havstad KM, Cushing J, Tweedie C, Fuentes O, Villanueva-Rosales N. Harnessing the power of big data: infusing the scientific method with machine learning to transform ecology. Ecosphere. 2014; 5: 67. doi: 10.1890/ES13-00359.1

[58] Dujon AM, Schofield G. Importance of machine learning for enhancing ecological studies using information-rich imagery. Endang Species Res. 2019; 39: 91–104. doi: 10.3354/esr00958

[59] Recknagel F. Applications of machine learning to ecological modelling. Ecol Modell. 2001; 146: 303–310. doi: 10.1016/S0304-3800(01)00316-7

[60] Thessen AE. Adoption of machine learning techniques in ecology and earth science. One Ecosyst. 2016; 1: e8621. doi: 10.3897/oneeco.1.e8621

[61] Miller-Coleman RL, Dodsworth JA, Ross CA, Shock EL, Williams AJ, Hartnett HE, et al. *Korarchaeota* diversity, biogeography, and abundance in Yellowstone and Great Basin Hot Springs and ecological niche modeling based on machine learning. PLoS ONE. 2012; 7: e35964. doi: 10.1371/journal.pone.0035964 22574130 PMC3344838

[62] Liu Z, Peng C, Work T, Candau JN, DesRochers A, Kneeshaw D. Application of machine-learning methods in forest ecology: recent progress and future challenges. Environ Rev. 2018; 26: 1–12. doi: 10.1139/er-2018-0034

[63] Tabak MA, Norouzzadeh MS, Wolfson DW, Sweeney SJ, Vercauteren KC, Snow NP et al. Machine learning to classify animal species in camera trap images: Applications in ecology. Methods Ecol Evol. 2018; 10: 585–590. doi: 10.1111/2041-210X.13120

[64] Yu P, Gao R, Zhang D, Liu ZP. Predicting coastal algal blooms with environmental factors by machine learning methods. Ecol Indic. 2021; 123: 107334. doi: 10.1016/j.ecolind.2020.107334

[65] Hedley JD, Mumby PJ. A remote sensing method for resolving depth and subpixel composition of aquatic benthos. Limnol Oceanogr. 2003; 48: 480–488. doi: 10.4319/lo.2003.48.1_part_2.0480

[66] Hochberg EJ, Atkinson MJ. Capabilities of remote sensors to classify coral, algae, and sand as pure and mixed spectra. Remote Sens Environ. 2003; 85: 174–189. doi: 10.1016/S0034-4257(02)00202-X

[67] Karpouzli E, Malthus TJ, Place CJ. Hyperspectral discrimination of coral reef benthic communities in the western Caribbean. Coral Reefs. 2004; 23: 141–151. doi: 10.1007/s00338-003-0363-9

[68] Joyce KE, Phinn SR, Roelfsema CM. Live coral cover index testing and application with hyperspectral airborne image data. Remote Sens. 2013; 5: 6116–6137. doi: 10.3390/rs5116116

[69] Parsons M, Bratanov D, Gaston K, Gonzalez F. UAVs, Hyperspectral Remote Sensing, and Machine Learning Revolutionizing Reef Monitoring. Sensors. 2018; 18: 2026. doi: 10.3390/s18072026 29941801 PMC6069449

[70] Andréfouët S, Payri C, Hochberg EJ, Hu C, Atkinson MJ, Muller-Karger FE. Use of *in situ* and airborne reflectance for scaling-up spectral discrimination of coral reef macroalgae from species to communities. Mar Ecol Prog Ser. 2004; 283: 161–177.

[71] Thompson DR, Hochberg EJ, Asner GP, Green RO, Knapp DE, Gao BC, et al. Airborne mapping of benthic reflectance spectra with Bayesian linear mixtures. Remote Sens Environ. 2017; 200: 18–30. doi: 10.1016/j.rse.2017.07.030

[72] Barott K, Smith J, Dinsdale E, Hatay M, Sandin S, Rohwer F. Hyperspectral and physiological analyses of coral-algal interactions. PLoS ONE. 2009; 4: e8043. doi: 10.1371/journal.pone.0008043 19956632 PMC2778555

[73] Pettersen R, Johnsen G, Bruheim P, Andreassen T. Development of hyperspectral imaging as a bio-optical taxonomic tool for pigmented marine organisms. Org Divers Evol. 2014; 14: 237–246. doi: 10.1007/s13127-013-0163-1

[74] Chennu A, Färber P, De’ath G, de Beer D, Fabricius KE. A diver-operated hyperspectral imaging and topographic surveying system for automated mapping of benthic habitats. Sci Rep. 2017; 7: 7122. doi: 10.1038/s41598-017-07337-y 28769060 PMC5541065

[75] Petropoulos GP, Arvanitis K, Sigrimis N. Hyperion hyperspectral imagery analysis combined with machine learning classifiers for land use/cover mapping. Expert Syst Appl. 2012; 39: 3800–3809. doi: 10.1016/j.eswa.2011.09.083

[76] Roberts CM, McClean CJ, Veron JEN, Hawkins JP, Allen GR, McAllister DE, et al. Marine biodiversity hotspots and conservation priorities for tropical reefs. Science. 2002; 295: 1280–1284. doi: 10.1126/science.1067728 11847338

[77] Mills MS, Deinhart ME, Heagy MN, Schils T. Small tropical islands as hotspots of crustose calcifying red algal diversity and endemism. Front Mar Sci. 2022; 9: 898308. doi: 10.3389/fmars.2022.898308

[78] Lobban CS, Tsuda RT. Revised checklist of benthic marine macroalgae and seagrasses of Guam and Micronesia. Micronesica. 2003; 35: 54–99.

[79] Randall RH. An annotated checklist of hydrozoan and scleractinian corals collected from Guam and other Mariana Islands. Micronesica. 2003; 35–36: 121–137.

[80] Paulay G. Marine biodiversity of Guam and the Marianas: Overview. Micronesica. 2003; 35–36: 3–25.

[81] van Beukering P, Haider W, Longland M, Cesar H, Sablan J, Shjegstad S, et al. The economic value of Guam’s coral reefs. Guam: University of Guam Marine Laboratory Technical Report 116; 2007.

[82] Burdick D, Brown V, Asher J, Caballes C, Gawel M, Goldman L, et al. Status of the coral reef ecosystems of Guam. Guam: Bureau of Statistics and Plans, Guam Coastal Management Program; 2008.

[83] Raymundo LJ, Burdick D, Lapacek VA, Miller R, Brown V. Anomalous temperatures and extreme tides: Guam staghorn *Acropora* succumb to a double threat. Mar Ecol Prog Ser. 2017; 564: 47–55. doi: 10.3354/meps12005

[84] Raymundo LJ, Burdick D, Hoot WC, Miller RM, Brown V, Reynolds T, et al. Successive bleaching events cause mass coral mortality in Guam, Micronesia. Coral Reefs. 2019; 38: 677–700. doi: 10.1007/s00338-019-01836-2

[85] Paulay G, Benayahu Y. Patterns and consequences of coral bleaching in Micronesia (Majuro and Guam) in 1992–1994. Micronesica. 1999; 31: 109–124.

[86] Reynolds T, Burdick D, Houk P, Raymundo L. Unprecedented coral bleaching across the Marianas Archipelago. Coral Reefs. 2014; 33: 499. doi: 10.1007/s00338-014-1139-0

[87] Mills MS, Ungermann M, Rigot G, den Haan J, Leon JX, Schils T. Assessment of the utility of underwater hyperspectral imaging for surveying and monitoring coral reef ecosystems. Sci Rep. 2023; 13: 21103. doi: 10.1038/s41598-023-48263-6 38036628 PMC10689744

[88] Obura DO, Aeby G, Amornthammarong N, Appeltans W, Bax N, Bishop J, et al. Coral reef monitoring, reef assessment technologies, and ecosystem-based management. Front Mar Sci. 2019; 6: 580. doi: 10.3389/fmars.2019.00580

[89] Shannon CE. A mathematical theory of communication. Bell Syst Tech J. 1948; 27: 379–423. doi: 10.1002/j.1538-7305.1948.tb01338.x

[90] Richards JA. Supervised classification techniques. In: Remote Sensing Digital Image Analysis: Springer Berlin, Heidelberg; 2013. pp. 247–318.

[91] Zhong Z, Li J, Luo Z, Chapman M. Spectral-Spatial Residual Network for hyperspectral image classification: A 3-D deep learning framework. IEEE Trans Geosci Remote. 2018; 56: 847–858. doi: 10.1109/TGRS.2017.2755542

[92] He K, Zhang X, Ren S, Sun J. Identity mappings in deep residual networks. In: Computer Vision-ECCV 2016: 14th European Conference, Amsterdam, The Netherlands, Proceedings Part IV: Springer International Publishing; 2016. pp. 630–345.

[93] Li Y, Yin B, Wang P, Zhang R. Non-intrusive load monitoring based on convolutional neural network mixed residual unit. J Phys: Conf Ser 2019; 1176: 022052. doi: 10.1088/1742-6596/1176/2/022052

[94] Oksanen J, Simpson GL, Blanchet FG, Kindt R, Legendre P, Minchin PR, et al. vegan: Community Ecology Package. R package version 2.6–4; 2022. Available from: https://CRAN.R-project.org/package=vegan

[95] Wickham H, Francois R, Henry L, Müller K. dplyr: A Grammar of Data Manipulation. R package version 1.0.10; 2022. Available from: https://CRAN.R-project.org/package=dplyr

[96] Buttigieg PL, Ramette A. A guide to statistical analysis in microbial ecology: a community-focused, living review of multivariate data analyses. FEMS Microbiol Ecol. 2014; 90: 543–550. doi: 10.1111/1574-6941.12437 25314312

[97] O’Brien JM, Scheibling RE. Turf wars: Competition between foundation and turf-forming species on temperate and tropical reefs and its role in regime shifts. Mar Ecol Prog Ser. 2018; 590: 1–17. doi: 10.3354/meps12530

[98] Graham NAJ, Chong-Seng KM, Huchery C, Januchowski-Hartley FA, Nash KL. Coral reef community composition in the context of disturbance history on the Great Barrier Reef, Australia. PLoS ONE. 2014; 9: e101204. doi: 10.1371/journal.pone.0101204 24983747 PMC4077760

[99] Adjeroud M, Kayal M, Iborra-Cantonnet C, Vercelloni J, Bosserelle P, Liao V, et al. Recovery of coral assemblages despite acute and recurrent disturbances on a South Central Pacific reef. Sci Rep. 2018; 8: 9680. doi: 10.1038/s41598-018-27891-3 29946062 PMC6018695

[100] Patterson Edward JK, Matthews G, Diraviya Raj K, Laju RL, Selva Bharath M, Arasamuthu A, et al. Coral mortality in the Gulf of Mannar, southeastern India, due to bleaching caused by elevated sea temperature in 2016. Curr Sci. 2018; 114: 1967–1972. doi: 10.18520/cs/v114/i09/1967-1972

[101] Estrada-Saldívar N, Jordán-Dahlgren E, Rodríguez-Martínez RE, Perry C, Alvarez-Filip L. Functional consequences of the long-term decline of reef-building corals in the Caribbean: evidence of across-reef functional convergence. R Soc Open Sci. 2019; 6: 190298. doi: 10.1098/rsos.190298 31824686 PMC6837220

[102] Vargas-Ángel B, Huntington B, Brainard RE, Venegas R, Oliver T, Barkley H, et al. El Niño-associated catastrophic coral mortality at Jarvis Island, central Equatorial Pacific. Coral Reefs. 2019; 38: 731–741. doi: 10.1007/s00338-019-01838-0

[103] Tkachenko KS, Huan NH, Thanh NH, Britayev TA. Extensive coral reef decline in Nha Trang Bay, Vietnam: *Acanthaster planci* outbreak: the final event in a sequence of chronic disturbances. Mar Freshw Res. 2020; 72: 186–199. doi: 10.1071/MF20005

[104] Bessel-Browne P, Epstein HE, Hall N, Buerger P, Berry K. Severe heat stress resulted in high coral mortality on Maldivian reefs following the 2015–2016 El Niño event. Oceans. 2021; 2: 233–245. doi: 10.3390/oceans2010014

[105] Adey WH. Coral reefs: Algal structured and mediated ecosystems in shallow, turbulent, alkaline waters. J Phycol. 1998; 34: 393–406. doi: 10.1046/j.1529-8817.1998.340393.x

[106] Tebben J, Motti CA, Siboni N, Tapiolas DM, Negri AP, Schupp PJ, et al. Chemical mediation of coral larval settlement by crustose coralline algae. Sci Rep. 2015; 5: 10803. doi: 10.1038/srep10803 26042834 PMC4650656

[107] Vargas-Ángel B, Richards CL, Vroom PS, Price NN, Schils T, Young CW, et al. Baseline assessment of net calcium carbonate accretion rates on U.S. Pacific reefs. PLoS ONE. 2015; 10: e0142196. doi: 10.1371/journal.pone.0142196 26641885 PMC4671731

[108] O’Leary JK, Barry JP, Gabrielson PW, Rogers-Bennett L, Potts DC, Palumbi SR, et al. Calcifying algae maintain settlement cues to larval abalone following algal exposure to extreme ocean acidification. Sci Rep. 2017; 7: 5774. doi: 10.1038/s41598-017-05502-x 28720836 PMC5515930

[109] Deinhart M, Mills MS, Schils T. Community assessment of crustose calcifying red algae as coral recruitment substrates. PLoS ONE. 2022; 17: e0271438. doi: 10.1371/journal.pone.0271438 35867665 PMC9307205

[110] Eckrich CE, Engel MS. Coral overgrowth by an encrusting red alga (*Ramicrusta* sp.) overgrowing scleractinian corals, gorgonians, a hydrocoral, sponges, and other algae in Lac Bay, Bonaire, Dutch Caribbean. Coral Reefs. 2013; 32: 81–84. doi: 10.1007/s00338-012-0961-5

[111] Nieder C, Chen PC, Chen CA, Liu SL. New record of the encrusting alga *Ramicrusta textilis* overgrowing corals in the lagoon of Dongsha Atoll, South China Sea. Bull Mar Sci. 2019; 95: 459–462. doi: 10.5343/bms.2019.0010

[112] Mills MS, Schils T. The habitat-modifying red alga *Ramicrusta* on Pacific reefs: a new generic record for the Tropical Northwestern Pacific and the description of four new species from Guam. PLoS ONE. 2021; 16: e0259336. doi: 10.1371/journal.pone.0259336 34780513 PMC8592442

[113] Stockton L, Edmunds PJ. Spatially aggressive peyssonnelid algal crusts (PAC) constrain coral recruitment to Diadema grazing halos on a shallow Caribbean reef. J Exp Mar Biol Ecol. 2021; 541: 151569. doi: 10.1016/j.jembe.2021.151569

[114] Burdick D. Guam coastal atlas: Providing benthic habitat data and other coastal information for the nearshore waters of Guam. University of Guam Marine Laboratory Technical Report 114; 2005.

[115] Mills MS, Schils T, Olds AD, Leon JX. Structural complexity of coral reefs in Guam, Mariana Islands. 2023; Remote Sens. 2023; 15: 5558. doi: 10.3390/rs15235558

[116] Dudgeon SR, Aronson RB, Bruno JF, Precht WF. Phase shifts and stable states on coral reefs. Mar Ecol Prog Ser. 2010; 413: 201–216. doi: 10.3354/meps08751

[117] Schürholz D, Chennu A. Digitizing the coral reef: Machine learning of underwater spectral images enables dense taxonomic mapping of benthic habitats. Meth Ecol Evol. 2023; 14: 596–613. doi: 10.1111/2041-210X.14029

[118] Albano PG, Sabelli B, Bouchet P. The challenge of small and rare species in marine biodiversity surveys: Microgastropod diversity in a complex coastal environment. Biodivers Conserv. 2011; 20: 3223–3237. doi: 10.1007/s10531-011-0117-x

[119] Pelletier D, Selmaoui-Folcher N, Bockel T, Schohn T. A regionally scalable habitat typology for assessing benthic habitats and fish communities: Application to New Caledonia reefs and lagoons. Ecol Evol. 2020; 10: 7021–7049. doi: 10.1002/ece3.6405 32760509 PMC7391553

[120] Cybulski JD, Husa SM, Duprey NN, Mamo BL, Tsang TPN, Yasuhara M, et al. Coral reef diversity losses in China’s Greater Bay Area were driven by regional stressors. Sci Adv. 2020; 6: eabb1046. doi: 10.1126/sciadv.abb1046 33008908 PMC7852383

[121] Donovan MK, Burkepile DE, Kratochwill C, Shlesinger T, Sully S, Oliver TA, et al. Local conditions magnify coral loss after marine heatwaves. Science. 2021; 372: 977–980. doi: 10.1126/science.abd9464 34045353

[122] Tegdan J, Ekehaug S, Hansen IM, Sandvik Aas LM, Steen KJ, Pettersen R, et al. Underwater hyperspectral imaging for environmental mapping and monitoring of seabed habitats. OCEANS 2015 –Genova, Genova, Italy. 2015; pp. 1–6. doi: 10.1109/OCEANS-Genova.2015.7271703

[123] Johnsen G, Ludvigsen M, Sørensen A, Sandvik Aas LM. The use of underwater hyperspectral imaging deployed on remotely operated vehicles–methods and applications. IFAC-PapersOnLine. 2016; 49: 476–481. doi: 10.1016/j.ifacol.2016.10.451

[124] Riera E, Hubas C, Ungermann M, Rigot G, Pey A, Francour P, et al. Artificial reef effectiveness changes among types as revealed by underwater hyperspectral imagery. Restor Ecol. 2023; e13978. doi: 10.1111/rec.13978

